# Editorial: Obesity across the life course

**DOI:** 10.3389/fpubh.2025.1591010

**Published:** 2025-06-13

**Authors:** Laura A. Gray, Anagha Killedar, Penny R. Breeze

**Affiliations:** ^1^Sheffield Centre for Health and Related Research, The University of Sheffield, Sheffield, United Kingdom; ^2^School of Public Health, Faculty of Medicine and Health, University of Sydney, Sydney, NSW, Australia

**Keywords:** obesity, life course, epidemiology, aging, obesity measurement

Obesity is a major health concern in many countries around the world. Coupled with an aging population, there are increasing numbers of patients who live with obesity alongside various co-morbidities. Obesity at different stages of the life course can have different influences, causes and consequences. Age is one of the biggest factors that influences obesity and persists from childhood into adolescence and adulthood (1, 2). This means it is important to investigate how obesity changes throughout the lifecourse.

In the first article of this Research Topic, Ulloque-Badaracco et al. carried out a systematic review investigating the association between obesity and vitamin B12, folate, and homocysteine levels in children and adolescents. The authors found no evidence of an association between obesity and vitamin B12 or folate, but found a statistically significant difference in homocysteine levels in children and adolescents who had obesity, compared to those who did not. A similar association has been found in a previous systematic review in adults (3) but this study shows that this association is apparent earlier in the life course. The authors suggest a number of potential reasons for the relationship between homocysteine levels and the presence of obesity; these are reduced absorption of nutrients due to poor diet (4), changes to gut microbiota, and how homocysteine is metabolized again due to poor diet (5), homocysteine insulin resistance due to impaired insulin sensitivity (6) affecting homocysteine metabolism regulation (7) or finally, inflammation and gut microbiota alterations influencing the breakdown of homocysteine (8, 9).

The second article by Hu et al., explored associations between BMI and waist circumference and premature mortality in young and middle-aged adults. This observational study used data from a large number of US individuals between 18 and 50 years old with robustness checks using a large cohort of Chinese individuals. They found a linear relationship between waist circumference and all-cause mortality, a relationship that was similar in men and women. A non-linear relationship between BMI and all-cause mortality was found, in accordance with the literature finding that both a high and low BMI is associated with premature mortality. Support for the obesity paradox (10), in middle-aged individuals, is provided by the finding that the risk of premature death decreased with increasing BMI in females aged 36-50 with a BMI below 28.6 kg/m^2^. This study highlights the differences in these relationships during young adulthood and middle age, illustrating the importance of taking a life course approach when studying obesity.

In the third article of this Research Topic, Fong et al. investigated lower-intensity weight management interventions for postnatal women delivered by non-specialists, exploring their effectiveness and implementation using a mixed-methods systematic review. They found that interventions had varied results with only three out of seven leading to a consistent reduction in weight in the intervention group and most interventions showing no or mixed effects. The authors conclude that there is a need for more research into this type of intervention, particularly investigating longer postnatal follow up periods. Research on lower-intensity interventions was lacking compared to that showing the effectiveness of higher-intensity postnatal weight loss interventions (11–13). This article also highlights the need for research into the women's perspective in such interventions. These perspectives are likely to change throughout the lifecourse and are particularly important to investigate in vulnerable life stages such as postpartum. The postpartum period represents a sensitive window of opportunity for the mother and infant and an example where it is necessary to take a life course approach in the study of obesity. In particular, this article suggests that interventions should be initiated within the first 6 months postpartum, suggesting that the timing of interventions during particular life stages is important.

The final article in this Research Topic, by Rogers et al., investigated the impact of a low-touch, self-guided and digital behavioral-based weight loss intervention on older adults. The study found that this intervention produced significant weight loss over the study period (20 weeks) and that this effect was greater in males than in females. Similar to other studies (14, 15), they found that increased engagement with the intervention was associated with great weight loss. The authors point out that this study uses weight, rather than body composition, and that body composition may be more clinically important for older adults due to the contributions of lean and fat mass to an individual's body weight (16).

The four articles in this Research Topic underline the need to study obesity across the lifecourse and that interventions should be specifically targeted at specific life stages due to differing causes and health consequences of obesity. The causes, consequences and confounders of obesity can vary at different life stages. Specifically, Ulloque-Badaracco et al. found evidence for a difference in homocysteine levels in children or adolescents with or without obesity, Hu et al. found the relationship between BMI and mortality differed between young and middle aged adults, Fong et al. stressed the need for timely interventions specific to certain life stages and Rogers et al. emphasized the importance of using an appropriate measure of body composition for the life stage of the subjects being studied. Against the wider research background, which has recently led to the development of different definitions and identification of obesity and overweight (17–19), this Research Topic of articles highlights the need to consider how we measure and define obesity so as to appropriately capture health risks across the lifecourse. BMI was not initially intended for use at an individual level, only for population surveillance (20), and the articles in this Research Topic highlight that we could assess obesity-related risk differently. Specifically, there was no association between BMI and B12 or folate (Ulloque-Badaracco et al.), premature mortality (Hu et al.) or participation in weight loss intervention for postnatal (Fong et al.) or older women (Rogers et al.). This might suggest that we could assess obesity-related health differently and that how we should measure obesity may vary at different life stages. BMI has often been used as a “risk-factor” for other health conditions, but we know that in certain populations it is not a good measure of obesity, particularly in older adults (18, 21) and people with athletic builds (18, 22). To more accurately explore the associations between adiposity and health, we need to consider using more appropriate and strict criteria for defining obesity, such as those recently proposed by an international collaboration of experts in diabetes and endocrinology (18). Further research is needed to explore how using such criteria in epidemiological research impacts the strength of associations between obesity and health problems, and how this might change at different stages of the life course.
